# A modular kernel approach for integrative analysis of protein domain boundaries

**DOI:** 10.1186/1471-2164-10-S3-S21

**Published:** 2009-12-03

**Authors:** Paul D Yoo, Bing Bing Zhou, Albert Y Zomaya

**Affiliations:** 1 Advanced Networks Research Group, School of Information Technologies (J12), the University of Sydney, NSW 2006, Australia; 2 Sydney Bioinformatics Centre and the Centre for Mathematical Biology, the University of Sydney, Sydney, NSW 2006, Australia

## Abstract

**Background:**

In this paper, we introduce a novel inter-range interaction integrated approach for protein domain boundary prediction. It involves (1) the design of modular kernel algorithm, which is able to effectively exploit the information of non-local interactions in amino acids, and (2) the development of a novel profile that can provide suitable information to the algorithm. One of the key features of this profiling technique is the use of multiple structural alignments of remote homologues to create an extended sequence profile and combines the structural information with suitable chemical information that plays an important role in protein stability. This profile can capture the sequence characteristics of an entire structural superfamily and extend a range of profiles generated from sequence similarity alone.

**Results:**

Our novel profile that combines homology information with hydrophobicity from SARAH1 scale was successful in providing more structural and chemical information. In addition, the modular approach adopted in our algorithm proved to be effective in capturing information from non-local interactions. Our approach achieved 82.1%, 50.9% and 31.5% accuracies for one-domain, two-domain, and three- and more domain proteins respectively.

**Conclusion:**

The experimental results in this study are encouraging, however, more work is need to extend it to a broader range of applications. We are currently developing a novel interactive (human in the loop) profiling that can provide information from more distantly related homology. This approach will further enhance the current study.

## Background

The accurate delineation of protein domain boundaries is an important step for the prediction of protein structure, function, evolution and design. Since a single domain spans an entire polypeptide chain or a subunit of such a chain, domains provide one of the most useful sources of information for understanding protein function, analysis based on domain families, and the study of individual proteins [1,2].

Proteins are composed of smaller building blocks, which are called "domains" or "modules". These building blocks are distinct regions in three-dimensional (3D) structure resulting in protein architectures assembled from modular segments that have evolved independently [3]. The modular nature of proteins has many advantages, offering new cooperative functions and enhanced stability. For example, new proteins, such as chimeric proteins, can be created because they are composed of multi-functional domains [4]. The search method for templates used in comparative modelling can be optimised by delineating domain boundaries, since the templates are classified on the basis of domains [5]. Domain boundary prediction can improve the performance of threading methods by enhancing their signal-to-noise ratio [6], and for homologous domains plays a key role in reliable multiple sequence alignment [7].

Over the past three decades, a large number of methods using the 3D coordinates of protein structure have been proposed for more accurately delineating domain boundaries [8]. However, the demand for fully automated approaches to identify domains in globular proteins from one-dimensional (1D) atomic coordinates has significantly grown over recent years [9,10]; because, genome and other sequencing projects have produced a flux of DNA and protein sequence data [11]. Many automated systems have shown reasonable improvements since they have successfully captured the information of a single molecule or of neighbouring residues involving short-range (local) interactions. However, at the same time, their limitations in the exploitation of information from long-range (non-local) interactions have been observed [12-15]. These limitations are related to model overfitting, and the weak signal-to-noise ratio associated with non-local interactions, which lead to the problem of the "vanishing gradient".

In this paper, we introduce a novel inter-range interaction integrated approach for protein domain boundary prediction. It involves (1) the design of modular kernel algorithm, which is able to effectively exploit the information of non-local interactions, and (2) the development of a novel profile that can provide suitable information to the algorithm. One of the key features of this profiling technique is the use of multiple structural alignments of remote homologues to create extended sequence profiles and combines the structural information with suitable chemical information that plays an important role in protein stability. This profile can capture the sequence characteristics of an entire structural superfamily and extend a range of profiles generated from sequence similarity alone.

## Results

To see the suitability of our proposed approach in domain boundary prediction, we have chosen the most widely adopted machine-learning models and profiles for comparison. Our experiment has three consecutive steps. First, we compare the performance of our modular neural network, Hierarchical Mixture of Experts (HME) with two other well-regarded machine-learning models in protein domain boundary prediction, transductive support vector machine (SVM) and multi-layered perceptron (MLP). Second, in the model comparison, the effectiveness of hydrophobicity information presented in Evolutionary and Hydrophobicity profile (EH-profile) is thoroughly tested and compared with widely known evolutionary profile, position specific scoring matrix (PSSM) generated by PSI-BLAST [16]. Last, the performance of our modular kernel approach (MKA) that consists of HME model and EH-profile is compared with three other protein domain boundary predictors on Benchmark_3 and CASP8 datasets.

The performance of each model was measured by the fractions of true negative and true positive (TN_f_: the proportion of true negative data correctly predicted) and TP_f_: the proportion of true positive data correctly predicted), the sensitivity (Sn: the proportion of correctly predicted domain boundary residues with respect to the total positively identified residues), the specificity (Sp: the proportion of incorrectly predicted boundary residues with respect to the total number of domain boundary residues), correlation-coefficient (Cc: an equal balance between positive and negative predictions, between -1 and 1) and accuracy (Ac: the proportion of true-positive and true-negative residues with respect to the total positive and negative residues). Cc reflects a situation in that a method, which predicts every residue to be positive, shows a prediction accuracy of 100% in detecting positive boundaries, but 0% accuracy for negative residues. Hence, a high value of Cc means that the model is regarded as a more robust prediction system.

We adopted a sevenfold cross-validation scheme for the model evaluation. Cross validation effectively solves the potential problems caused by residual evaluations. Because the residual uses the entire dataset in the training, it does not give an indication of how well the model will predict for unseen data. For this reason, we remove some of the data before training begins. When training is completed, the data that was removed can be used to test the performance of the learned model on new data. The advantage of this method is that it matters less how the data gets divided. Every data point gets to be in one of the test sets (exactly once), and gets to be in one of the training sets (six times in sevenfold cross validation).

In this experiment, the dataset is divided into seven subsets, and the holdout method is repeated seven times. Each time, one of the seven subsets is used as the test set and the other (six) subsets are put together to form a training set. The estimated prediction accuracy is the average of the prediction accuracy for the models, derived from the independently and randomly generated test divisions.

In our preliminary experiments [17], we tested five different window sizes (3, 7, 11, 19 and 27) for each model and found that the window size of 11 is the most suitable for our experiments. A window size of 11 means which contain 23 amino acids with 11 preceding and 11 following amino acids for the boundary residue (located at the centre of the window).

Table 1 summarises confusion matrices for each test model. The predictive performance of proposed model (HME) was compared with two other machine-learning models. They were tested with two different profiles. As indicated, the standard deviation for each model is insignificant, suggesting reasonable performance consistency. The average accuracy over three models for EH-profile is about 3 percentage points better than evolutionary profile. This proves our hypothesis that the hydrophobicity information used in EH-profile provides suitable information as it performs key roles for protein stability. Clearly, EH-profile is more useful than the widely known evolutionary profile for protein domain boundary prediction. More importantly, the performance of HME with EH-profile showed the best predictive performance (Ac: 0.78). With evolutionary profile, it also outperformed other models in Sn, Cc, and Ac. The modular approach used in HME improved its predictive performance by effectively capturing the information from non-local interactions. In other words, it is more resistant to model overfitting, and the weak signal-to-noise ratio associated, which lead to the problem of vanishing gradient.

**Table 1:** Predictive performance of machine-learning models

Models	**TN**_f_	**TP**_f_	Sensitivity (Sn)	Specificity (Sp)	Correlation- Coefficient (Cc)	Accuracy (Ac)
HME_HE_	0.77 ± 0.015	0.79 ± 0.026	0.78 ± 0.002	0.78 ± 0.012	0.56 ± 0.016	0.78 ± 0.015
HME_PSSM_	0.74 ± 0.019	0.74 ± 0.018	0.75 ± 0.010	0.73 ± 0.045	0.48 ± 0.023	0.74 ± 0.016
SVM_HE_	0.71 ± 0.008	0.73 ± 0.010	0.70 ± 0.003	0.74 ± 0.017	0.44 ± 0.011	0.72 ± 0.020
SVM_PSSM_	0.71 ± 0.004	0.67 ± 0.008	0.65 ± 0.012	0.72 ± 0.006	0.37 ± 0.007	0.69 ± 0.003
MLP_HE_	0.69 ± 0.009	0.72 ± 0.012	0.61 ± 0.027	0.75 ± 0.019	0.40 ± 0.013	0.70 ± 0.025
MLP_PSSM_	0.67 ± 0.017	0.71 ± 0.032	0.61 ± 0.013	0.76 ± 0.027	0.37 ± 0.022	0.68 ± 0.011

Mean testing data ± standard deviation obtained using ANOVA test using optimal settings for each model.

Finally, our modular kernel approach (MKA) that comprises HME model and EH-profile, and three other well-known predictors, were evaluated on Benchmark_3 and CASP8 datasets. DOMpro [18] uses evolutionary information (gene-exon shuffling), secondary structure and solvent accessibility information with a recursive neural network. DOMpro is trained and tested on a curated dataset derived from the CATH database. It achieved a sensitivity and specificity of 71% and 71%, respectively in the CAFASP4 and was ranked among the top *ab initio *domain predictors. DomNet [17] is a recently introduced machine-learning algorithm that uses a novel compact domain profile (CD-profile). It outperformed nine other machine-learning methods on Benchmark_2 dataset. DomNet is trained with an inter-domain linker-region index, secondary structure and relative solvent accessibility information with CD-profile. CD-profile uses additional structural information from conserved-domain database [19] because conserved domains contain conserved sequence patterns or motifs, which allows for their detection in polypeptide sequences. Hence, the PSSMs in conserved domain database can be useful to find remote homology. DomPred [20] uses a combined homology and fold-recognition based approach. The sequence homology approach simply attempts to distinguish boundaries from overlapping edges in PSI-BLAST multiple sequence alignments using hidden Markov models. The fold recognition approach relies on secondary structure element alignments, using DomSSEA method [20] to find domain boundaries in more distant homologs. The DomSSEA has been shown to provide a rapid prediction of the fold for given sequences with no detectable homology to any known structure and have also been applied to the related problem of novel fold detection. The method has an accuracy of 49% at predicting the domain boundary location within 20 residues using a representative set of two domain chains.

Table 2 shows the accuracies obtained by each predictor on Benchmark_3 and CASP8 datasets. The accuracies were provided based on the number of domains in a sequence. We combined multi-domain proteins in CASP8 dataset. This, because, we have too small number of sequences (causing statistically insignificant) if we split them based on the number of domains.

**Table 2:** Accuracy of domain boundary placement on the Benchmark_3 dataset and the CASP8

Predictors	B_3 1-domain	B_3 2-domain	B_3 3-domain	CASP8 single-domain	CASP8 multi-domain
MKA	82.1%	50.9%	31.5%	86.4%	51.9%
DomNet	83.0%	54.3%	21.0%	84.7%	46.8%
DOMpro	79.2%	48.1%	29.8%	75.1%	40.6%
DomPred	86.7%	9.5%	31.7%	87.4%	29.2%

Benchmark_3: 1-domain (39.1%), 2-domain (39.9%), and 3-domain and more (21%); CASP8: single-domain (68.6%), and multi-domail (31.4%).

MKA correctly predicted 86 of all 106 targets for 1-domain chains and showed 82.1% accuracy. The accuracy of MKA was 0.9 percentage points less than DomNet in 1-domain prediction. In 2-domain prediction, DomNet still performed better as it predicted 113 of all 208 chains correctly. However, with 3-domain and more chains, only MKA correctly predicted with above 30% accuracy. Its accuracy in this category was 10.5 and 11.7 percentage points higher than DomNet and DOMpro respectively. Again, MKA showed the best performance (51.9%) with the multi-domain proteins in CASP8. This means MKA more consistently captures information from EH-profile and eventually leads to model stability and robustness. Although it is well acknowledged that the model stability is a more important factor than the learning bias in predictive performance [21], several important issues that should be taken into account in order to improve the performance of the proposed MKA. This will be discussed in the next section.

Two experiments performed in this study proved that hydrophobicity information presented in the EH-profile provides useful information. However, the PSSMs in the conserved domain database used by DomNet can be of a central source, providing valuable structural/homology information. Because, conserved sequence patterns in the PSSMs of conserved domain database are effectively recognised by its learning model. The computational learning model was also specially designed for processing high-dimensional data with the focus of the exploitation of local-interaction information. Because of these capacities, the predictor showed even more powerful performance in the prediction of single-domain proteins and as demonstrated in the two above-mentioned experimental results.

## Discussion

Although many machine-learning-based domain predictors have been developed, they have shown limited capability for multi-domain proteins. Our approaches used in MKA were shown to be effective for multi-domain proteins. The two experiments confirmed our hypothesis that MKA efficiently captures non-local interaction information while preserving accurate data modelling in domain-boundary prediction. However, as its prediction accuracy reaches only about 40% for multi-domain and 82% for one-domain proteins, there is still much room for improvement. Some areas of possible improvement are discussed in this section.

### Non-local interactions in amino acids

As historical summaries have shown [22], many researchers have built successful secondary structure predictors using machine learners such as feed-forward neural networks and support vector machines with local input windows of 9-15 amino acids [23-26]. Over the years, the performance has steadily improved by about one percent per year. This was possible because of increased training data and several additional techniques including (1) output filers to cleanup predictions, (2) input profiles - associated with homologous sequence alignments and (3) predictor ensembles. The main weakness of these approaches resides in the researchers' use of a local window that cannot capture non-local information such as that presented in β-sheets. This is partially corroborated because the β-sheet class always shows the weakest performance results. Substantially increasing the input window's size, however, does not seem to improve the performance. As long as we cannot fully capture information about the interaction of remote sequence positions, efficient learning for the long-range dependencies does not appear possible. The learner is given only a set of inputs and a serial order relation for them and must solve a difficult credit assignment problem to identify the interacting positions.

**Figure 1:**
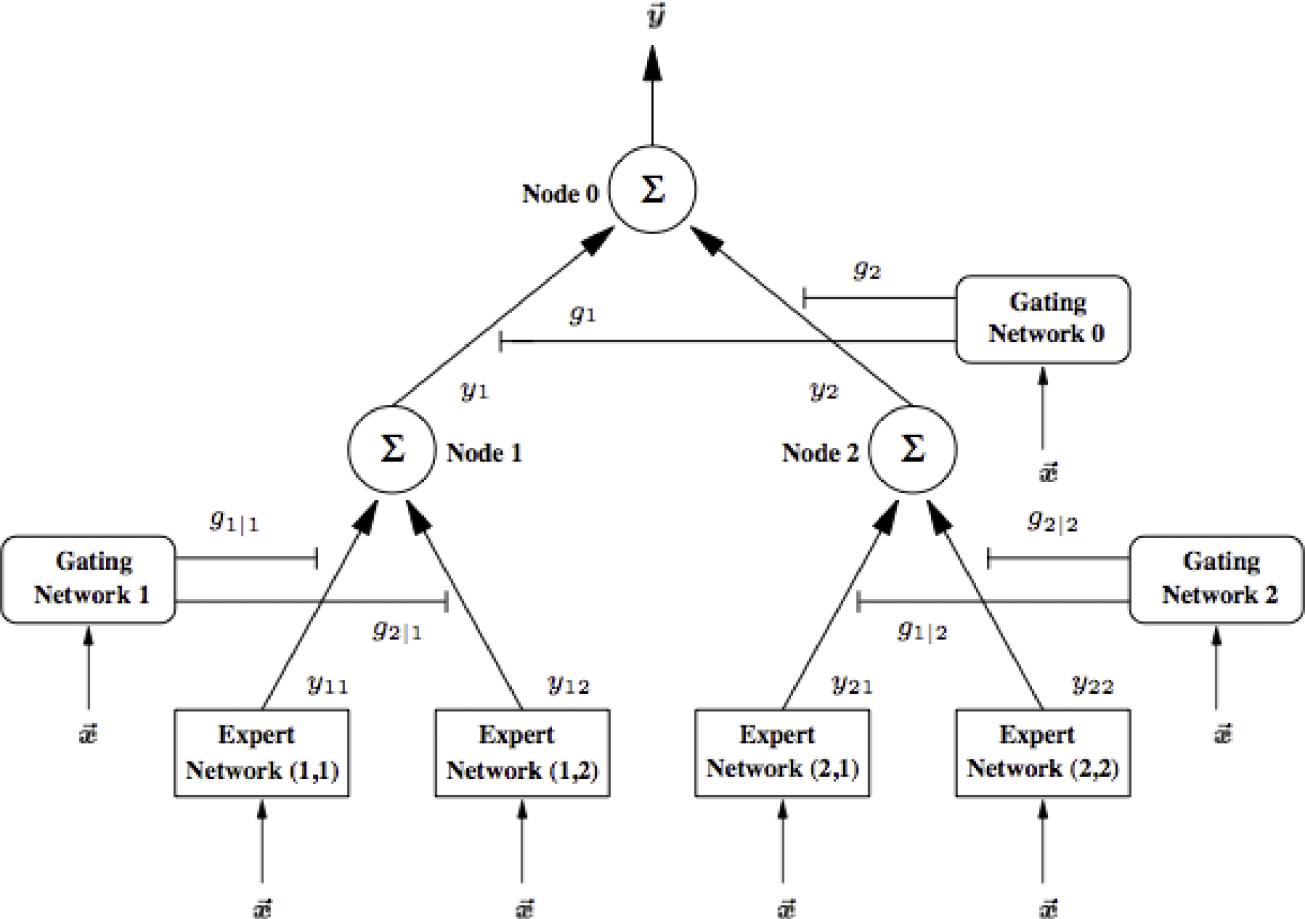
**A basic architecture of Hierarchical mixture of experts network**.

Our modular kernel approach using HME architecture consists of comparatively simple experts (specialists neural) and gating networks, organised in a tree structure (Figure 1). The basic functional principle behind this structure is the well-known technique called "divide and conquer". This technique solves complex problems by dividing them into simpler problems for which solutions can be obtained easily. These partial solutions are then integrated to yield an overall solution to the whole problem. Its architecture enforces constant error flow (thus, neither exploding nor vanishing) through internal states of units.

Many gradient-based machine learners solve their classification problem (i.e. function approximation) by explicitly hard splitting the input space into sub-regions, such that only one single "expert" is contributing to the overall output of the model. The "hard splits" of the input space make algorithms to be variance increasing, especially in the case of higher dimensional input spaces where data is very sparsely distributed. In contrast, HME architecture uses a soft splitting approach to partition the input space instead of hard splitting, as is the case in statistical models, allowing the input data to be present simultaneously in multiple sub-regions. In this case, many experts may contribute to the overall output of the network, which has a variance decreasing effect.

### Secondary structure information

In the literature, protein secondary-structure information has been widely used for domain-boundary prediction, as it was shown to be useful for increasing prediction accuracy. Most inter-domain regions are composed of loops while β-strands tend to form sheets that constitute the core of protein domains. The α-helices and β-sheets in proteins are relatively rigid units and therefore domain boundaries rarely split these secondary structure elements. The mutations at the sequence level can obscure the similarity between homologs. However, their secondary-structure patterns remain more conserved because changes at the structural level are less tolerated. The secondary-structure-alignment methods used in this study aim to exploit these conserved features to locate domain regions within secondary-structure strings. We obtained the secondary-structure information by one of the widely known secondary-structure predictors called SSpro [27]. However, there is one significant limitation: the best predictor still cannot reach the upper boundary of prediction accuracy. The best secondary-structure predictors show only about 75-80% accuracy. Clearly, the incorrectly predicted secondary structures are highly likely to lead to the incorrect delineation of domain boundaries. Although the predicted secondary information seems to be useful for current approaches, it may not be ideal if one attempts to reach better than 80% accuracy.

### Hydrophobicity and profiles

One of the existing powerful methods for rapidly shifting through protein data is homology modelling, which uses dynamic-programming-alignment methods to search evolutionarily related (and hence similar) sequences in the databases of known sequences. In the last decade, a number of machine-learning-based systems have used evolutionary profiles that contain homology information from sequence alignments and showed striking improvements [17,23-25,28-31]. This has been a major breakthrough in protein structure prediction literature [23]. This profiling technique that provides suitable information for the base algorithm opened a way on how to effectively incorporate valuable information into computational structure prediction models.

For prediction or classification tasks, it is well-known that finding the right features or information plays key roles in improving model performance. Our profiling method based on the assumption that hydrophobicity, a major factor in protein stability with a suitable homology information can provide better information for its computational leaner proved to be successful. However, many more issues need to be investigated, as indicated in various alignments studies [32-35]. One of the examples is human intervention in the adjustment of automatic alignment. As widely believed, domain expert intervention at (1) fold identification and (2) readjustments multiple alignment levels can significantly improve its accuracy. In the literature, therefore, research to develop more biologically realistic profiles has been actively reported. This should prevent the current limitations of automated methods by allowing domain experts to interact with the computation to control the quality of analysis at processing stages.

### Domain assignment is more accurate for proteins with fewer domains

In general, the prediction accuracy of sequence-based methods has been far smaller (< 50%) for multi-domain proteins. For example, Liu and Rost's [36] experiments on CATH and SCOP assigned domains to random subsets of 1187 proteins of known high-resolution structure and less than 10% sequence homology; they showed correct prediction of the number of domains (single and multi) in 69% of the cases. However, the accuracy for multi-domain cases alone was only 38%. For the two continuous-domain proteins, the average accuracy of *dbp *prediction in different validation runs was 46-51% considering a prediction to be correct if it were in ± 20 residues interval of the CATH- and SCOP-assigned boundaries.

Joshi [37] discussed the main reasons for the problems in deciphering the multi-domain-protein structures and his possible solutions. With experimental data, although the structure within a domain is fixed, the relative positioning of two domains within the same chain can vary. For this reason, and because protein structural domains are independent folding units, it is unusual to find single crystal structures containing more than one domain. Similarly, protein modelling by database searching, sequence alignment and/or phylogenic analysis is better performed on a single domain rather than a multi-domain polypeptide. Hence, in most cases, the number of domains in a protein should first be identified to determine the locations of such domains on the primary chain before embarking on a standard method of protein-structure/function determination. The identification of linker regions connecting two distinct domains is also useful in finding domain-boundary locations; accordingly, several domain-boundary predictors employing domain-linker information, such as DomCut and DomainDiscovery, showed reasonably better predictive performance in domain-boundary prediction.

### Continuous vs. discontinuous

Since Wetlaufer [38] introduced the classification of domains into continuous and discontinuous, a large number of researches have been done in these separate fields. Continuous domains form from a single-chain segment whereas discontinuous domains are composed of two or more chain segments. The boundary prediction for discontinuous domains remains very difficult, especially from *ab initio *approaches. The most current and successful *ab initio *method for predicting discontinuous domains is SnapDRAGON [39]. It is comparably reliable and as it requires a set of homologous sequences, similar to the target sequence to generate a multiple sequence alignment as input. It showed the accuracy for continuous domains is 63.9% while only 35.4% for discontinuous domains, with an overall accuracy of 51.8%.

The main reason for poor performance in case of discontinuous domains appears to be that use of secondary elements is not appropriate in such cases [40]. A component of (sequentially) local organisation is partly an element in the domain but is not sufficient as some domains are formed from segments of the protein sequence that are distant in the primary chain. The β-sheet also influences the definition of a domain since β-sheets are rarely split into separate domains. However, although one sheet would not normally be in two domains, two or more sheets might be in one domain, so again this structural element does not provide a sufficient definition.

The partitioning of the structure into domains may result in domains consisting of continuous stretches of ploypeptide chain, one stretch per domain (continuous domains). Frequently, however, regions of the polypeptide chain that are distant in sequence, are close together in 3D structure, thus a domain may consist of two or more segments of the chain, which are non-continuous in sequence (discontinuous domains). Our method simply assigns each domain segment to a separate domain, ignoring at this time the relatively rare problem of non-continuous domains.

## Conclusion

We have firstly used modular kernel approach (MKA) in protein domain boundary prediction as a novel method to effectively tackle the problem of non-local interaction. Our approach adopted modular HME that leverages evolutionary and hydrophobicity information in the form of profiles and also used predicted secondary structure and relative solvent accessibility. This was demonstrated in the three consecutive experiments in this study. The novel EH-profile that combines homology information with hydrophobicity from the SARAH1 scale was successful in providing more structural and chemical information. In addition, the modular approach adopted in HME proved to be effective in capturing information from non-local interactions. Each memory-based model in HME (Figure 1) showed a learning ability to bridge time intervals at some level in the non-local interaction environment (This is the case of noisy and incompressible input sequences), without much loss of a short-time-lag capability (the time interval in the learning process between residues involving non-local interactions). With Benchmark_3 and CASP8 datasets, our approach showed its usefulness, especially in the case of multi-domain chains.

## Methods

Our approach to domain boundary prediction consists of three consecutive steps. (1) comprehensive multi-domain dataset construction for the purpose of benchmarking structure-based domain identification methods. (2) novel evolutionary and hydrophobicity profile design and (3) the construction of modular neural network for the exploitation of non-local interaction information.

### Multi-domain benchmark dataset

Benchmark_3 is a newly developed comprehensive dataset for benchmarking structure-based domain-identification methods. Benchmark_3 is similar to the dataset published by Holland et al. [41]; it contains proteins of known structures for which three methods - e.g. CATH [42] and SCOP [43] - agree on the assignment of the number of domains. The dataset consists of 271 polypeptide chains, 106 one-domain chains (39.1%), 108 two-domain chains (39.9%), 45 three-domain chains (16.6%), 7 four-domain chains (2.6%) and 5 five-domain chains (1.9%). Also, 44 chains were removed from the Benchmark_2, as the overlap between the domains was below 90%. The dataset is nonredundant in a structural sense: each combination of topologies occurs only once per dataset. Sequences of protein chains are taken from the Protein Data Bank (PDB) [44]. The secondary-structure information and solvent accessibility are predicted for each chain in Benchmark_3, using SSpro [27] and ACCpro [45]. Evolutionary information for each chain is obtained using the position-specific scoring matrix (PSSM), which was constructed using PSI-BLAST [16].

CASP8 is the latest benchmark dataset in the Critical Assessment of Techniques for Protein Structure Prediction (CASP) competition. Annually, most of the well-known domain predictors participate in the CASP competition. Further information on the available datasets is at http://predictioncenter.org/casp8/. The dataset contains 88 single-domain chains and 40 multi-domain chains. For each chain, we obtained secondary-structure information, solvent accessibility, PSSM and inter-domain linker index, using the previously mentioned methods.

### Evolutionary and hydrophobicity profile

The existing models of multiple sequence alignments are generally represented by sequence patterns proving homology information (e.g. consensus sequences [46]). This has been regarded as one of the most valuable information for determining local protein structures. To construct more informative profile, EH-profile uses one effective hydrophobicity scale in addition to evolutionary information generated by PSI-BLAST.

A number of researchers selected hydrophobicity as the main feature among many other physicochemical properties for protein structure prediction (such as polarity, charge or size) [47-49]. Several recent studies reported that the level of phosphorylation affect protein's hydrophobicity significantly or vice versa [50,51]. Hydrophobicity is a major factor in protein stability. The "hydrophobic effect" plays a fundamental role in the spontaneous folding of proteins. It can be expressed as the free energy (kilocalories per mole) of transfer of amino-acid side-chains from cyclohexane to water. The amino acids with positive values of free energy in transferring cyclohexane to water are hydrophobic, and the ones with negative values are hydrophilic [47]. Table 3 shows hydrophobicity scales, and the hydrophobicity matrix can be formulated using the following function.

**Table 3:** Hydrophobicity scale: nonpolar → polar distributions of amino-acids chains, pH7 (kcal/mol)

	Amino acid	Feature value		Amino acid	Feature value
1	I	4.92	11	Y	--0.14
2	L	4.92	12	T	--2.57
3	V	4.04	13	S	--3.40
4	P	4.04	14	H	--4.66
5	F	2.98	15	Q	--5.54
6	M	2.35	16	K	--5.55
7	W	2.33	17	N	--6.64
8	A	1.81	18	E	--6.81
9	C	1.28	19	D	--8.72
10	G	0.94	20	R	--14.92

Given:

Amino_Acid [] = {C, Q, E, G, H, I, L, K, M, F, P, S, T, W, Y, V, ...} and

Hydrophobicity_Index [] = {1.28, -5.54, -6.81, 0.94, -4.66, 4.92, 4.92, -5.55, 2.35, 2.98, 4.04, -3.40, -2.57, 2.33, -0.14, 4.04, ...},

where the denominator 20 is used to convert the data range into [0,1].

Hydrophobicity matrix [3,4] means the absolute value of the difference of the hydrophobicity indices of two amino acids E (-6.81) and G (0.94). With the range adjustment, we obtain 0.2935.

In the case of structure/function families and the classification of protein sequences, various hydrophobicity scales were thoroughly examined by David [52]. He showed the effectiveness of numerous hydrophobicity scales, and concluded that the Rose scale [53] was superior to all others when used for protein structure prediction. The Rose scale is correlated to the average area of buried amino acids in globular proteins (Table 4). However, Korenberg et al. [54] pointed out several key drawbacks with Rose scale. Since it is not a one-to-one mapping, different amino-acid sequences can have identical hydrophobicity profiles; the scale covers a narrow range of values while causing some amino acids to be weighted more heavily than others. To overcome these problems, the SARAH1 scale - five bits "state" representation for amino acid - was introduced by Korenberg et al.

**Table 4:** Rose hydrophobicity scale

	Amino acid	Feature value		Amino acid	Feature value
1	A	0.74	11	L	0.85
2	R	0.64	12	K	0.52
3	N	0.63	13	M	0.85
4	D	0.62	14	F	0.88
5	C	0.91	15	P	0.64
6	Q	0.62	16	S	0.66
7	E	0.62	17	T	0.70
8	G	0.72	18	W	0.85
9	H	0.78	19	Y	0.76
10	I	0.88	20	V	0.86

SARAH1 assigns each amino acid a unique five-bit signed code, where exactly two bits are non-zero. SARAH1 ranks 20 possible amino acids according to the Rose hydrophobicity scale (Table 4). Each amino acid is assigned a five-bit code in descending order of the binary value of the corresponding code. One of the benefits of using the five-bit code is that the complexity of the classifier can be significantly reduced and can arrange these numbers in 32 possible ways (2^5 ^= 32). If the representations with no or all 1 s, and those with one or four 1 s are removed, there are exactly 20 representations left. This leaves just enough representation to code for the 20 amino acids. In the case of window-size 5, a residue vector has 5 × 11 = 55 dimensions, which leads to less model complexity than the residue vector using widely used orthogonal encoding (20 × 11 = 220 dimensions) [55].

The resulting scale in Table 5, where the right-half is the negative mirror image of the left-half, is referred to as SARAH1. The 10 most hydrophobic residues are positive, and the 10 least hydrophobic residues are negative. Korenberg et al. indicated that while the above scales carry information about hydrophobicity, scales can similarly be constructed to embed other chemical or physical properties of the amino acids such as polarity, charge, α-*helical *preference, and residue volume.

**Table 5:** SARAH1 Scale

	Amino acid	Binary code		Amino acid	Binary code
1	C	1,1,0,0,0	11	G	0,0,0,--1,--1
2	F	1,0,1,0,0	12	T	0,0, --1,0, --1
3	I	1,0,0,1,0	13	S	0,0, --1, --1,0
4	V	1,0,0,0,1	14	R	0, --1,0,0, --1
5	L	0,1,1,0,0	15	P	0, --1,0, --1,0
6	W	0,1,0,1,0	16	N	0, --1, --1,0,0
7	M	0,1,0,0,1	17	D	--1,0,0,0, --1
8	H	0,0,1,1,0	18	Q	--1,0,0, --1,0
9	Y	0,0,1,0,1	19	E	--1,0, --1,0,0
10	A	0,0,0,1,1	20	K	--1, --1,0,0,0

### Non-local interaction and vanishing gradient problem

In protein structure prediction problem, existing large kernel algorithms such as neural networks have performed well; however, they have also shown several limitations especially when dealing with non-local interactions in amino acids. The main difficulty with this class of neural networks is due to the lack of generally efficient algorithms for solving numerical optimisation. In particular, error minimisation is known to fail in the presence of non-local interactions [56,57]. Interesting remedies to this vanishing gradient have been suggested in the literature [58,59]; however, their effectiveness in realistically large scale supervised learning tasks has not been elucidated so far.

To overcome this limitation, one should be able to minimise the problem of the "vanishing gradient" [56,57]. In the case of non-local-interaction, residues that are close in space (3D-strucure) occupy distant positions in the sequence. At each sequence position the model may receive important structural information needed at distantly located sequences. Therefore, it must deal with long-term dependencies, which leads to the problem of the vanishing gradient. The vanishing gradient addresses the characteristics of non-chaotic dynamic systems that the gradient of states with respect to previous states vanishes exponentially with the temporal distance between these states. This feature of non-chaotic systems results from the fact that initial conditions do not have a large influence over later states. Therefore, non-chaotic systems are prevented from learning to store information over time.

### A modular approach to neural networks

This new modular approach to neural networks combines a number of methods and procedures to effectively exploit non-local information. The first step was to develop a modular kernel model and train it to predict domain boundaries of proteins with an EH-profile. Within this model, each kernel has a learning ability capable of bridging intervals of time so that even in the case of noisy, incompressible input sequences, without the loss of a short-time-lag capability. Its architecture enforces constant error flow (thus, neither exploding nor vanishing) through internal states of units. Being modular, this approach requires several small networks to cooperate and communicate with each other in order to obtain the complete map of inter-molecular interactions.

These networks are comprised of modules which can be categorised both according to their distinct structure and to their functionality, which are integrated together via an integrating unit. With functional categorisation, each module is a neural network, which carries out a distinct identifiable subtask. This approach allows different types of learning algorithms (these can be neural network based or otherwise) to be combined in a seamless fashion. Through the utilisation and integration of the best-suited learning algorithms for a given task, there is a distinct improvement in artificial neural network learning. As with other modular learning systems the main advantages include extendibility, incremental learning, continuous adaptation, economy of learning and re-learning, and computational efficiency.

### Hierarchical mixture of experts

This approach incorporates the Hierarchical Mixture of Experts (HME), a well-known tree-structured model for regression and classification based on soft probabilistic splits of the input space [60]. In this model, the distribution of target variables is given by a mixture of component distributions in which the components, as well as the mixing coefficients, are conditioned on the input variables. The component distributions are referred to as *experts*, while mixing coefficients are controlled by *gating distributions*. Values for the parameters of this model can be set using an efficient EM algorithm to predict maximum likelihood [60]. The resulting model will automatically perform a soft partitioning of the dataset into groups corresponding to different regions of input space and simultaneously fit separate models (corresponding to the mixture components) to each of those groups.

The fundamental concept behind the probabilistic interpretation of this network is that a paralinguistic mapping of input vectors *x*^(*t*) ^to output vectors *y*^(*t*) ^in the dataset can be subdivided into sequence of nested decisions, generating a probabilistic tree. For a particular input vector *x*^(*t*)^, values generated by the gating networks are assumed to be multinomial probabilities which select one of the connected expert networks. A sequence of decisions starts from the top node influenced by the probability distributions of the intermediate gating networks. The process eventually ends at a specific terminal expert network.

HME describes a conditional probability distribution over a vector *t *of target variables, conditioned on a vector *x *of inputs (Figure 1). Consider the case of functional mapping learning of the type  based on training data set T = (*x*^(*t*)^, *y*^(*t*)^), *t *= 0, ..., *n *with  and a corresponding desired response . All of the networks, both experts and gating, receive the same input vector at the *t*^*th *^time instant, *x*^(*t*)^. However, while the gating networks use this input to compute confidence level values for the outputs of the connected expert networks, the expert networks themselves use the input to generate an estimate of the desired output value. The outputs of the gating networks are scalar values and are a partition of unity at each point in the input space, i.e. a probability set. Thus, consider the two-layered binary branching HME as depicted in Figure 1: Each of the expert neural networks (*i*, *j*) produces outputs *y*_*ij *_from the input vector *x*^(*t*) ^according to the relationship:

where *f *is a neural network mapping using input *x*^(*t*) ^and its corresponding weight matrix . The input vector *x*^(*t*) ^is considered to have an additional constant value to allow for network bias. The gating networks are generally linear. Since they perform multi-directional classification among the expert networks, the non-linear output is chosen to be a "softmax" (short for soft maximum). The outputs of the gating network *g*_*i *_at the top level are computed according to:

where  is the weight matrix associated with gating network *g*_*i*_. Due to the special form of the softmax being non-linear, the *g*_*i*_'s are positive and sum up to one for each input vector *x*^(*t*)^. They can be interpreted as the local conditional probability in that an input vector *x*^(*t*) ^lies in the affiliated partitioned sub-region of the associated expert network. The lower level gating networks compute their output activations similar to the top level gating network according to the following expression:

The output activations of the expert networks are weighted by the gating networks' output activations as they proceed up the tree to form the overall output vector. Specifically, the output of the *i*^*th *^internal node in the second layer of the tree is:

while the output at the top level node is:

Since both the expert and the gating networks compute their activations as functions of the input , the overall output of the architecture is a non-linear function of the input.

### Overall architecture

Our modular approach contains three main components. First, given amino-acid sequences, PSI-BLAST was used to generate PSSMs with an e-value threshold, for the inclusion of 0.001 and six search iterations of nonredundant (*nr*) sequence database. The PSSM has 20 × *N *elements, where *N *is the length of the target sequence, and each element represents the log-likelihood of a particular residue substitution, based on a weighted average of BLOSUM62 [61] matrix scores for a given alignment position in the template. Second, SARAH1 scales were computed from the amino-acid chains in Benchmark_3 dataset and combined with the PSSM. The EH-profile, which contains PSSMs and SARAH1 scales, were all normalised to fall in the interval [-1, 1] using the following algorithm.

where *p *is *R *× *Q *matrix of input vectors, minp is *R *× *1 *vector containing minimums for each *p*, and *maxp *is *R *× *1 *vector containing maximums for each *p*.

Third, our modular kernel model used the resulting profile and performed its classification tasks. As discussed, we adopted a sevenfold cross-validation scheme for its evaluation. With the threshold *T*, the final predictions were simulated from the raw output generated by HME. During the post-processing of the network output, because the network generates raw outputs with many local peaks, we again adopted Liu and Rost's [36] method to filter the raw outputs. First, we determined the threshold for each network output according to the length (*L*) of the protein and to the distribution of raw output values for all residues in that protein. We compiled the 92^nd ^percentile of the raw output *T*_1 _and set the threshold *T *to:

*T *was set to the threshold that divides domain boundaries and others. If the value of a residue was above the threshold, the residue was regarded as domain boundary. Second, we assigned the central residue as a domain boundary if three or more residues were predicted as a domain boundary. And all parameters for these filters were developed using the validation set alone.

The performance of our modular approach was measured by accuracy (Ac), sensitivity (Sn) specificity (Sp), correlation coefficient (Cc), and the fractions of true negative (TN_f_) and true positive (TP_f_). The Sn, Sp, Ac and CC can be expressed in terms of true positive (TP), false negative (FN), true negative (TN) and false positive (FP) predictions.

and

**Figure 2:**
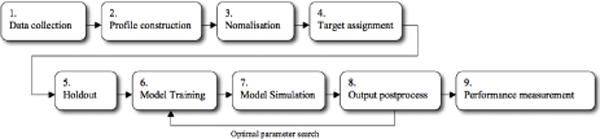
**The flowchart of MKA showing the stepwise procedure**. Figure 1 shows the stepwise procedure we have performed. (1) data collection, building Benchmark_3 and pre-processing datasets; (2) profile construction, such as PSSM, Sarah1 and EH-profile; (3) the information obtained in (2) and (3) were combined and normalised to fall in the interval [--1, 1] to be fed into networks; (4) target levels were assigned to each profile (positive, +1, for domain boundary residues and negative, --1, for non-boundary residues); (5) a hold-out method, to divide the combined dataset into seven subsets (training and testing sets); (6) model training on each set, to create a model; (7) simulation of each model on the test set, to obtain predicted outputs; and (8) post-processing to find predicted domain boundary locations. The procedure from (6) to (8) was performed iteratively until we obtained the most suitable kernel and the optimal hyperparameters for HME for Benchmark_3 dataset.

The flowchart of MKA showing the stepwise procedure we have performed is presented in Figure 2.

## List of abbreviations used

Ac: Accuracy; Cc: Correlation-coefficient; CD-profile: Compact domain profile; EH-profile:Evolutionary and hydrophobicity profile; HME: Hierarchical mixture of experts; MKA: Modular kernel approach: MLP: Multi-layered perceptron; NR: Nonredundant; PSI-BLAST: Position specific iterated basic local alignment search tool; PSSM: Position specific scoring matrix; SARAH1: Simultaneously axially and radially alignment hydrophobicity; SN: Sensitivity; SP: Specificity; SVM: Support vector machine; TN_f_: Fraction of true negative; TP_f_: Fraction of true positive; 1D: One-dimensional; 3D: Three-dimensional

## Competing interests

The authors declare that they have no competing interests.

## Authors' contributions

PDY developed and implemented the novel approaches, and drafted the manuscript. PDY prepared datasets, programming in MATLAB, C++, Shell and Perl scripts, and interpreted the results with BBZ. BBZ and AYZ edited the manuscript and introduced the problem initially.

## Note

Other papers from the meeting have been published as part of *BMC Bioinformatics *Volume 10 Supplement 15, 2009: Eighth International Conference on Bioinformatics (InCoB2009): Bioinformatics, available online at http://www.biomedcentral.com/1471-2105/10?issue=S15.
